# Senolytic Therapy as a Preventive Strategy for Spine Degeneration and Pain

**DOI:** 10.1002/advs.202522770

**Published:** 2026-05-25

**Authors:** Saber Ghazizadeh, Hosni Cherif, Matthew Mannarino, Juiena Sagir, Magali Millecamps, Jean A. Ouellet, Laura S. Stone, Lisbet Haglund

**Affiliations:** ^1^ Department of Surgery Orthopaedic Research Lab McGill University Montreal Quebec Canada; ^2^ Department of Surgery McGill Scoliosis and Spine Group McGill University Montreal Quebec Canada; ^3^ Alan Edwards Centre For Research on Pain (AECRP) McGill University Montreal Quebec Canada; ^4^ ABC‐platform (Animal Behavioral Characterization) At the Alan Edwards Centre for Research on Pain McGill University Montreal Quebec Canada; ^5^ Département de biomédecine vétérinaire Faculté de médecine vétérinaire Université De Montréal Saint‐Hyacinthe Quebec Canada; ^6^ Shriner's Hospital for Children Montreal Quebec Canada; ^7^ Department of Anesthesiology University of Minnesota Minneapolis Minnesota USA

**Keywords:** back pain, musculoskeletal health, senescence, senolytic therapy, SPARC‐null mice

## Abstract

Cellular senescence drives inflammation and tissue breakdown and is a key hallmark of aging. The accumulation of senescent cells is strongly linked to the degeneration of spinal tissues and back pain. Here, we show that administration of the senolytic agents, o‐vanillin and RG‐7112, prevents the development of pain‐related behavior in *sparc^−/−^* mice. Treated mice exhibit a reduced expression of senescence markers in the intervertebral discs, vertebral endplates, vertebral bone, and spinal cord, alongside a dampening of pro‐inflammatory senescence‐associated secretory factors in these tissues. Early senolytic intervention also preserved intervertebral disc volume and vertebral bone microarchitecture, indicating protection against structural spine degeneration. These findings demonstrate that targeting cellular senescence at an early stage can mitigate degenerative changes and pain, supporting senolytic therapy as a promising preventive strategy for musculoskeletal decline.

## Graphical abstract

Cellular senescence promotes inflammation, tissue degeneration, and chronic back pain. In sparc‐null mice, early oral administration of the senolytic agents o‐vanillin and RG‐7112 reduced senescent cell burden and pro‐inflammatory SASP signaling across intervertebral discs, endplates, vertebral bone, and spinal cord. Preventive senotherapy preserved disc volume and bone microarchitecture, dampened neuroinflammation, and effectively blocked the development of pain‐related behaviors, supporting senolytics as a strategy to delay spine degeneration.

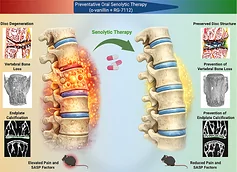

## 1 Introduction

Cellular senescence, a key hallmark of aging and degenerating tissues, is characterized by a stable and typically non‐reversible cell‐cycle arrest that develops in response to stressors such as DNA damage, oxidative stress, mitochondrial dysfunction, and epigenetic instability [1, 2]. Senescent cells (SnCs) accumulate in tissues over time and actively contribute to age‐related pathologies such as osteoarthritis [3, 4, 5], neurodegeneration [6, 7, 8], and cancer [9, 10, 11]. Beyond loss of proliferative potential, SnCs develop a pro‐inflammatory phenotype known as the senescence‐associated secretory phenotype (SASP) [9, 12]. The SASP is composed of cytokines, chemokines, growth factors, and matrix‐degrading enzymes [13, 14]. These molecules alter the surrounding tissue environment by impairing structural integrity and amplifying inflammation. Furthermore, it promotes paracrine senescence, thereby exacerbating tissue dysfunction. Due to their resistance to apoptosis, SnCs persist in tissues and act as active drivers of tissue deterioration [15, 16].

Identification of SnCs has relied on senescence‐associated β‐galactosidase (SA‐β‐Gal) lysosomal activity [17]; however, this marker lacks specificity, as elevated SA‐β‐Gal activity can occur in non‐senescent contexts, including certain immune, quiescent, or stressed cells [18]. More definitive indicators include the cyclin‐dependent kinase inhibitors *p21* and *p16^Ink4a^*, which are broadly accepted as key molecular markers of early and late senescence, respectively [19, 20, 21]. While *p21* is primarily induced by p53 and reflects transient or early senescence in response to acute stress [22], *p16^Ink4a^* tends to accumulate with age and marks a more stable, late‐stage senescent phenotype [20, 23]. These subtypes also differ in their SASP composition and apoptotic threshold, with cells expressing high *p16^Ink4a^* levels being more resistant to clearance [24]. Understanding this heterogeneity is crucial for designing senolytic strategies, as certain drugs preferentially target either *p16^Ink4a^* or *p21*‐expressing senescent cells, influencing therapeutic outcomes across diseases.

Pharmacological removal of senescent cells with senomorphic and senolytic agents has emerged as a promising therapeutic approach for delaying or reversing age‐related tissue deterioration [25, 26]. Drugs such as navitoclax (Bcl‐2 inhibitor), FOXO4‐DRI (p53 inhibitor), and RG‐7112 (MDM2 inhibitor) exemplify synthetic senolytic compounds. Natural compounds, including quercetin, fisetin, and o‐vanillin, exhibit both senolytic and senomorphic activity [3, 10, 25, 26, 27, 28, 29, 30, 31, 32, 33, 34, 35]. While senolytic drugs eliminate SnCs, senomorphic compounds have dual effects and, in addition to eliminating SnCs, suppress the detrimental effects of the SASP [32, 36].

Back pain, often associated with intervertebral disc (IVD) degeneration, is the leading cause of years lived with disability globally [34, 35, 37, 38]. The socioeconomic impact is substantial, with healthcare costs in the United States alone surpassing $100 billion annually [39, 40]. Degenerating IVDs display an elevated level of SnCs expressing SASP components such as IL‐1β, IL‐6, and matrix metalloproteinases [41, 42, 43, 44, 45]. These molecules contribute to extracellular matrix breakdown, neurovascular infiltration [46], and nociceptor sensitization [47], ultimately driving both structural failure and chronic pain [48].

In our previous study, we demonstrated that treatment with either o‐vanillin or RG‐7112 reduced SnCs and SASP factors in human IVD cells and tissues. These treatments improved matrix homeostasis and cell viability, indicating translational potential [49, 50, 51, 52, 53]. We have also previously demonstrated that o‐vanillin and RG‐7112 reduced the SnC burden, improved disc structure, suppressed inflammatory mediators, and attenuated behavioral signs of back pain in middle‐aged *sparc^−/−^* mice, which already had well‐established IVD degeneration and back pain when the treatment was initiated [53]. Although treatment improved most outcomes, it was not able to bring the levels back to those of healthy animals.

Building on our prior findings, this study investigates whether administration of o‐vanillin and RG‐7112 can prevent IVD degeneration and associated pain in *sparc^−/−^* mice if treatment is initiated at the time when back pain is emerging. In addition, given the distinct molecular targets of the agents, we assessed whether combination therapy would provide an enhanced benefit over single therapy. Our study was designed to evaluate the impact of preventive senotherapeutic administration on disc and bone health, inflammatory signaling, and pain behavior.

## 2 Results

### 2.1 Senotherapeutics Prevented the Development of a Pain Phenotype in *Sparc^−/−^* Mice

**FIGURE 1:**
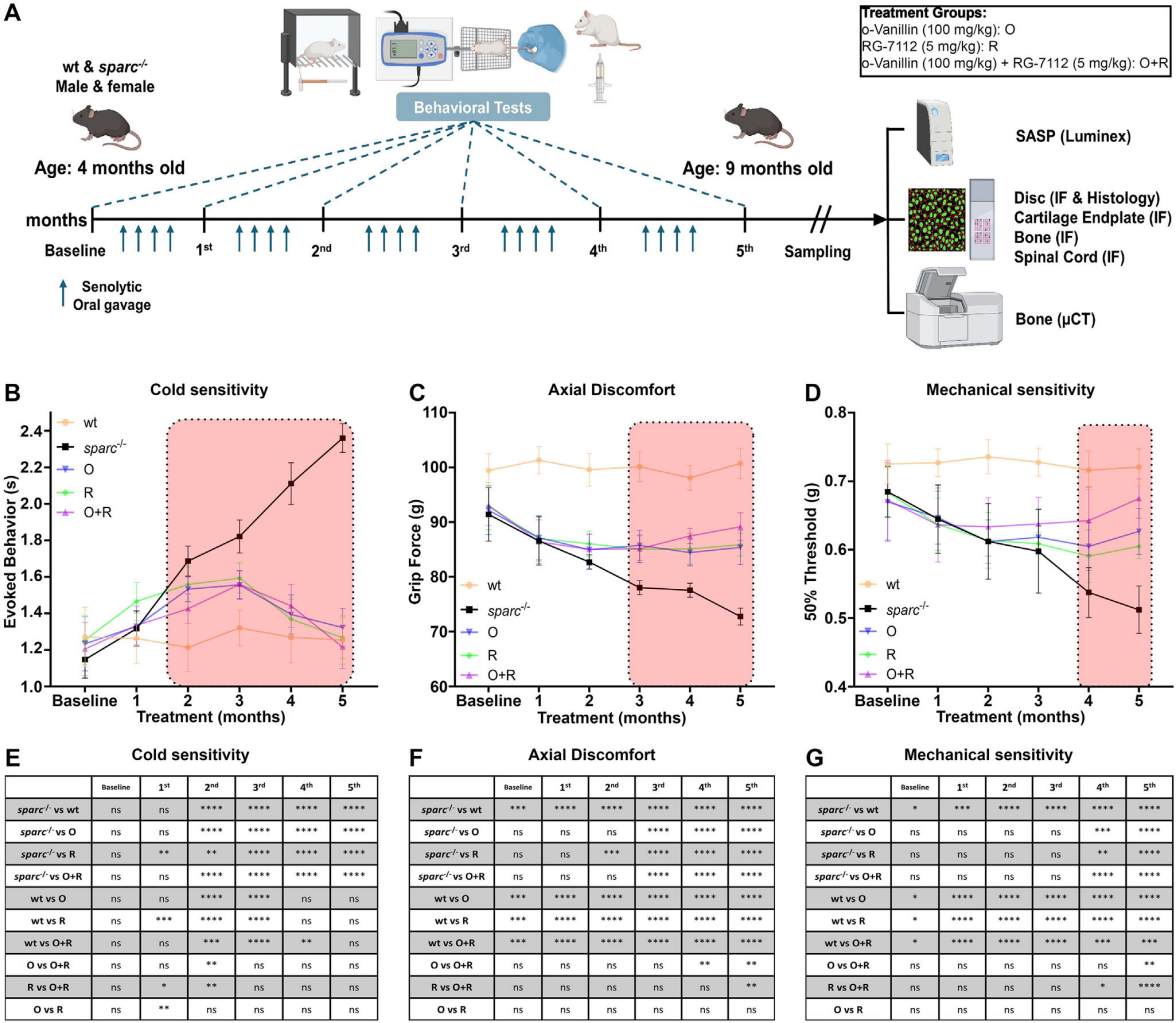
Preventive senolytic therapy attenuated pain‐related behavior in *sparc* ^−/−^ mice. (A) Experimental design and treatment groups. Male and female wt and *sparc^−/−^* mice were followed from 4 to 9 months and received weekly oral gavage of vehicle or senolytic treatment: o‐vanillin (O), RG‐7112 (R) or combination (O+R). Pain assessment tests were performed every month by (B) cold sensitivity, (C) axial discomfort and (D) mechanical sensitivity. Statistical comparisons between groups at each time point for (E) cold sensitivity, (F) grip strength, and (G) mechanical sensitivity tests. Data are represented as means ± SD and analyzed by two‐way ANOVA with Tukey's post hoc test. *n* = 15 animals per group (8 males and 7 females). *p < 0.05, **p < 0.01, ***p < 0.001, ****p < 0.0001 and ns not significant. µCT, Micro‐computed tomography; IF, immunofluorescence; SASP, senescence‐associated secretory phenotype.

*Sparc^−/−^* mice begin to show signs of back pain at 4 months of age, with pain becoming well‐established by 7 months [54, 55, 56]. To follow the onset and progression of pain, a comprehensive behavioral analysis was conducted once a month between 4 and 9 months of age, in both male and female mice. Experimental groups and treatment timelines are depicted in Figure 1A. *Sparc^−/−^* mice received weekly oral treatments with vehicle, o‐vanillin (100 mg/kg), RG‐7112 (5 mg/kg), or a combination (100 mg/kg + 5 mg/kg). Treatment dosages were based on earlier experiments conducted in 7 to 9‐ month old *sparc^−/−^* mice [53].

Grip strength was used to determine axial pain, while von Frey and acetone‐evoked behavior were used to evaluate radiating pain. Vehicle‐treated *sparc^−/−^* mice developed a progressively worsening pain phenotype across all tests, whereas wild‐type (wt) mice maintained a stable, non‐painful behavioral profile throughout the experimental period (Figure 1B–D and Figure S1A–C).

At baseline, no significant differences were detected between wt and *sparc^−/−^* mice in cold sensitivity (Figure 1B,E). Treatment with o‐vanillin or RG‐7112 attenuated the development of cold hypersensitivity, with significant effects observed after 2 months of o‐vanillin and 1 month of RG‐7112 treatment. After 4 months of o‐vanillin and 5 months of RG‐7112 treatment, cold sensitivity was not significantly different from that observed in wt animals. The therapeutic effect was not further improved by a combination treatment (Figure 1B,E).

A small but significant difference was detected at baseline between wt and *sparc^−/−^* mice in grip strength (Figure 1C,F). The decline in grip strength was prevented after 2 months and was significantly higher than vehicle‐treated *sparc^−/−^* mice after 3 months of o‐vanillin and 2 months of RG‐7112 treatment. After 5 months of treatment, combination therapy significantly improved grip strength compared to single treatment; however, values remained below the wt levels (Figure 1C,F).

A small but significant difference was also detected at baseline between wt and *sparc^−/−^* mice in mechanical sensitivity (Figure 1D,G). The mechanical sensitivity was attenuated after 3 months with single treatment and after 2 months with combination treatment and was significantly reduced after 4 months of treatment with either o‐vanillin, RG‐7112 or combination treatment, with combination therapy reaching wt‐equivalent thresholds after 4 months of treatment (Figure 1D,G). After 5 months of treatment, combination therapy significantly improved mechanical sensitivity compared to the drug administered as a single treatment (Figure 1D,G). We compared changes across all groups and time points for each behavioral assay, and observed no significant change in cold sensitivity, axial discomfort, and mechanical sensitivity in wt mice; a progressive worsening of these measures was observed in vehicle‐treated *sparc^−/−^* mice, and an attenuation of the temporal changes was observed following senolytic treatment (Figure S1A–C).

These findings show that treatment with o‐vanillin or RG‐7112 reduced pain‐related behaviors in *sparc* ^−/−^ mice, while a combination treatment further improved grip strength and reduces mechanical sensitivity. No difference in body weight or mortality was observed between the groups (Figure S1D–F). There were no significant differences between male and female animals.

### 2.2 Senotherapeutics Decreased SnCs in the Spinal Cord

**FIGURE 2:**
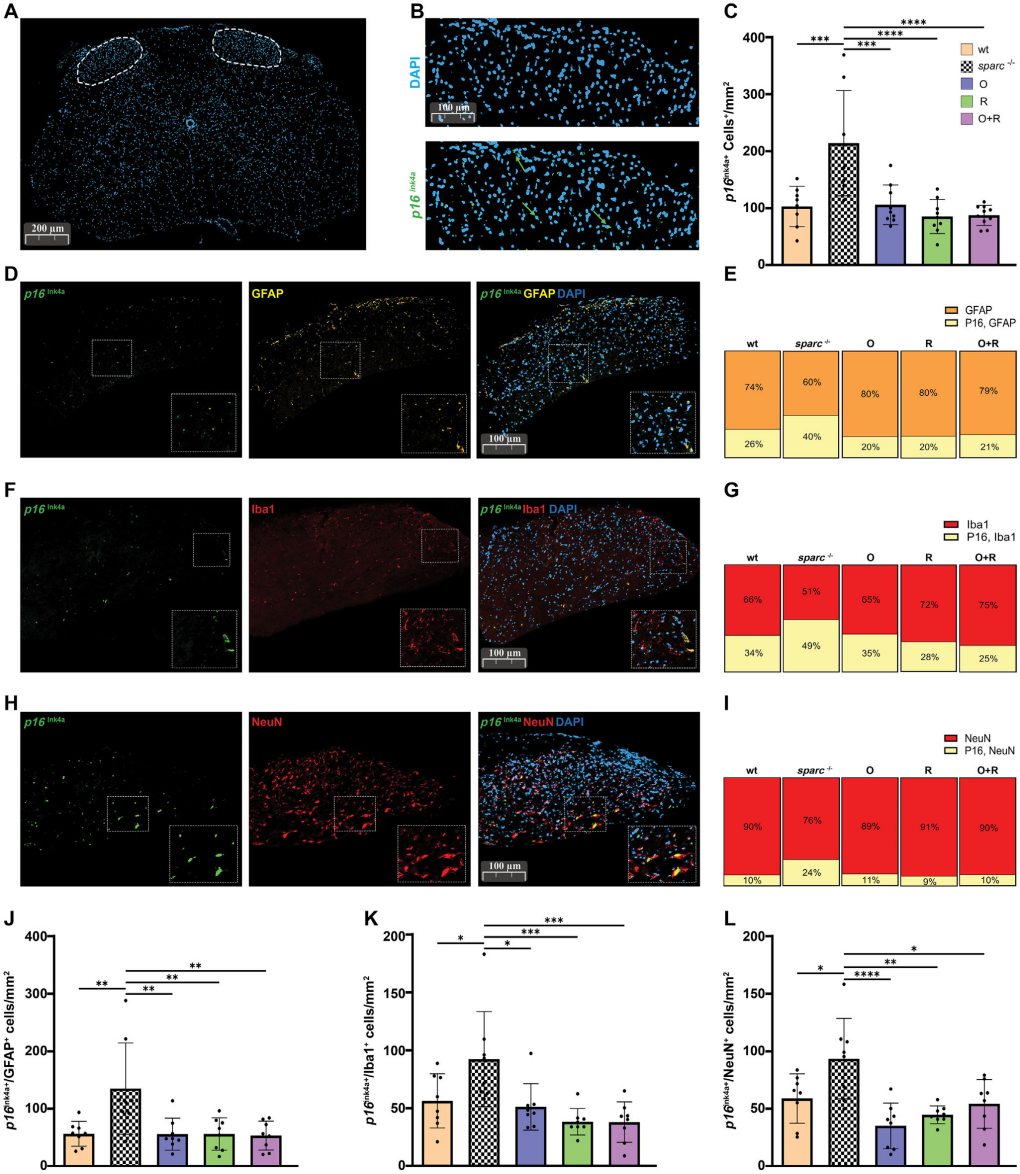
Senolytic treatment reduced senescent cells in the dorsal horn. (A) Representative image of a DAPI‐stained spinal cord section, with the dorsal horn region of interest (ROI) outlined by a dashed line and (B) *p16^Ink4a^* immunoreactivity in the dorsal horn. (C) Quantification of *p16^Ink4a^*‐positive cells in the dorsal horn. (D) Representative images of p*16^Ink4a^*, and GFAP expression. (E) Percentage of GFAP and GFAP/ *p16^Ink4a^* double‐labelled cells. (F) Representative images showing *p16^Ink4a^* and Iba1 expression. (G) Percentage of Iba1 and Iba1/ *p16^Ink4a^* double‐labelled cells. (H) Representative images showing *p16^Ink4a^*, and NeuN expression. (I) Percentage of NeuN and NeuN/ *p16^Ink4a^* double‐labelled cells. Quantification of (J) p*16^Ink4a^*/GFAP positive astrocytes, (K) *p16^Ink4a^*/Iba1 positive microglia, and (L) *p16^Ink4a^*/NeuN positive neurons, expressed as positive double‐labelled cells/mm^2^. DAPI served as a nuclear counterstain. For each animal, the mean of three independent images in the ROI was calculated for group analysis. n = 8‐10 animals per group (4 to 5 males or females). The scale bar in A represents 200 µm, whereas the scale bars in B, D, F, and H (merged images) represent 100 µm. Data are presented as means ± SD and were analyzed by an ordinary one‐way ANOVA followed by Tukey's post hoc test. *p < 0.05, **p < 0.01, ***p < 0.001, and ****p < 0.0001. No significance was observed between wt and drug‐treated groups or between the drug treatments.

The presence of SnCs in the central nervous system (CNS), including neural and glial populations, has been implicated in the development of chronic pain [57, 58]. We evaluated the effects in the dorsal horn, as it is the primary site of central processing, integration, and modulation of nociceptive information, where nociceptive signals from dorsal root ganglion (DRG) neurons are first integrated [59, 60, 61]. *Sparc^−/−^* mice showed elevated glial fibrillary acidic protein (GFAP), and cluster of differentiation molecule 11b (CD11b) immunoreactivity consistent with previously reported pain‑related spinal cord changes [62] and the known roles of astrocytes and glial activation in chronic pain [63, 64, 65, 66]. A significantly higher number of *p16^Ink4a^*‐positive cells was found in the dorsal horn of vehicle‐treated *sparc* ^−/−^ mice compared to the wt group, consistent with a previous report (Figure 2A‐C and Figure S2A). Treatment with o‐vanillin, RG‐7112 or their combination resulted in significantly fewer *p16^Ink4a^*‐positive cells compared to the vehicle‐treated *sparc* ^−/−^ group, and in all cases, levels were comparable to those observed in wt controls (Figure 2C). To further assess the role of senolytics in modulating pain phenotypes and to identify which cell populations were senescent, we colocalized the expression of *p16^Ink4a^* with GFAP, a marker of reactive astrocytes, ionized calcium‐binding adaptor molecule 1 (Iba1), a marker of activated microglia and neuronal nuclei (NeuN) a marker for mature and post‐mitotic neurons. S*parc* ^−/−^ mice displayed a higher number of senescent GFAP‐positive astrocytes (Figure 2D,E,J and Figure S2B), Iba1‐positive microglia (Figure 2F,G,K and Figure S2C) and NeuN‐positive neurons (Figure 2H,I,L and Figure S2D) per area compared to the wt group.

Senolytic treatment with either o‐vanillin or RG‐7112 significantly reduced the number of senescent GFAP‐positive astrocytes, Iba1‐positive microglia, and senescent NeuN‐positive neurons in the dorsal horn (Figure 2J–L). The combination did not provide an added effect compared to single drug treatment. Interestingly, all treatments restored levels to those observed in wt controls. The total number of *p16^Ink4a^* cells exceeded the number of cells identified through colocalization, indicating that additional cell types may contribute to the overall senescence burden.

### 2.3 Senotherapeutics Reduced SASP Factor Release from the IVDs

Lumbar degenerating IVDs from *sparc^−/−^* mice exhibit elevated secretion of SASP factors, which have been previously associated with back pain and disc degeneration [53]. In our prior study, we demonstrated that by 7 and 9 months of age, degenerating *sparc^−/−^* IVDs display increased expression of multiple SASP factors, and that therapeutic intervention with o‐vanillin and RG‐7112 attenuated the release of some [53]. However, it remains unknown whether early intervention can prevent the establishment of a senescence‐associated inflammatory microenvironment.

**FIGURE 3:**
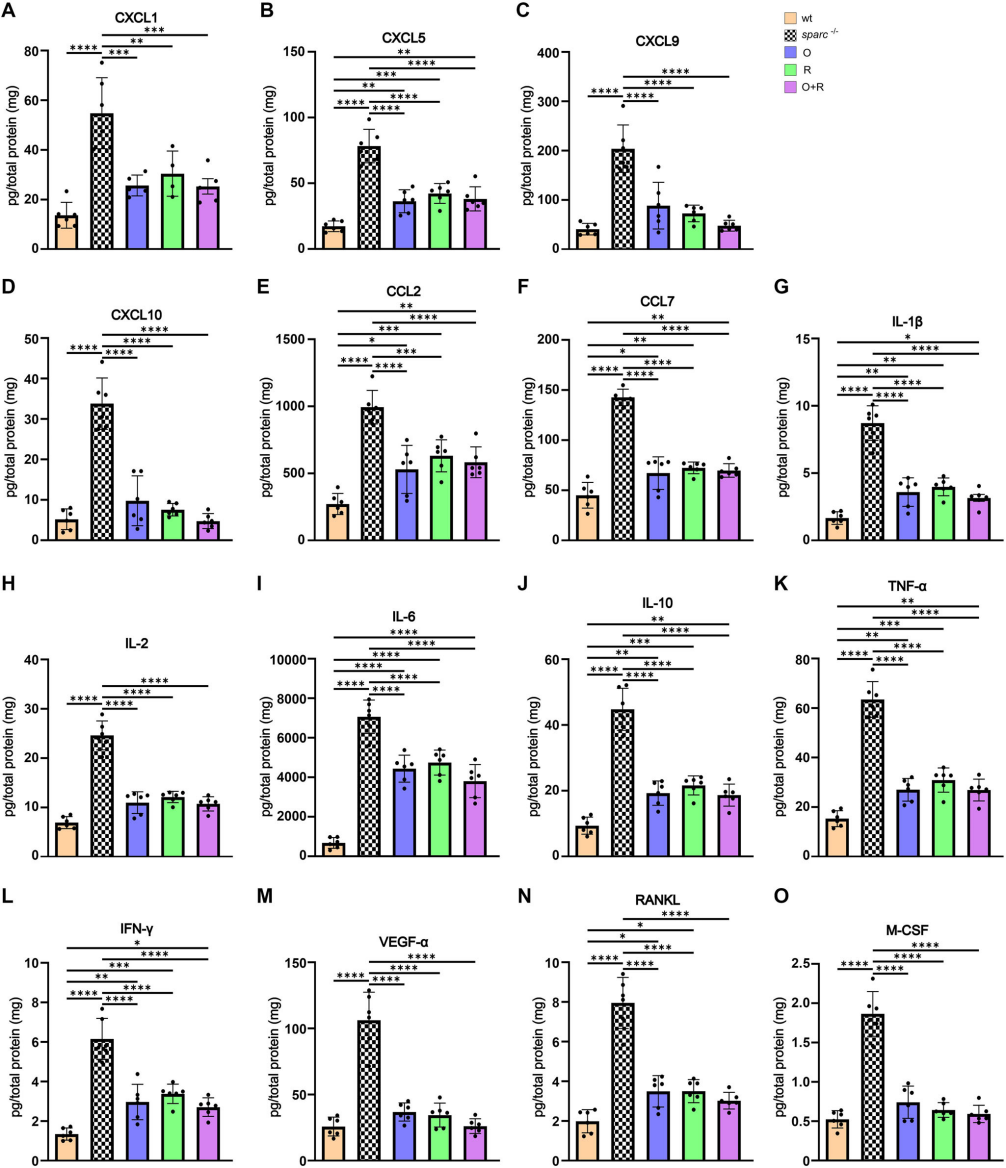
Senotherapeutic treatment decreased SASP factor secretion from the IVDs. (A–F) chemokines, (G–L) cytokines, and (M–O) growth factor release from IVDs were assessed by a multiplex assay. *n* = 6 animals (3 discs/animal), (3 males and 3 females) in each group. The data are presented as means ± SD; one‐way ANOVA followed by Tukey's post hoc comparison Tukey's were used to measure significant differences between the groups. The cytokine concentrations were normalized to total protein (pg/mg total protein). *p < 0.05, **p < 0.01, ***p < 0.001, and ****p < 0.0001.

To investigate the effect of preventive treatment on SASP factor release, lumbar IVDs of treated and untreated animals were harvested at the termination of the experiment. The lumbar region was selected due to its critical biomechanical role and its high susceptibility to age‐related degeneration and back pain in both humans and *sparc^−/−^* mice [55]. The secretion profiles of 15 SASP‐associated cytokines, chemokines and growth factors were quantified using a Luminex multiplex assay (Figure 3A–O).

Consistent with the previous study, vehicle‐treated lumbar *sparc^−/−^* IVDs exhibited significantly elevated levels of all 15 SASP factors compared to wt control. Treatment with either o‐vanillin, RG‐7112 or combination led to significant reductions in all 15 SASP factors relative to vehicle‐treated *sparc^−/−^* mice. Among the 15 SASP factors measured, following combination treatment, levels of nine proteins (CXCL1, CXCL9, CXCL10, CCL2, IL‐1β, IL‐2, VEGF‐α, RANKL, and M‐CSF), were not significantly different from those of wt controls, indicating restoration to wt levels. A summary of the average concentrations is provided in Table S1.

### 2.4 Senotherapeutics Prevented a Loss of Disc Volume and Reduced SnC Burden and Disc Degeneration Severity

**FIGURE 4:**
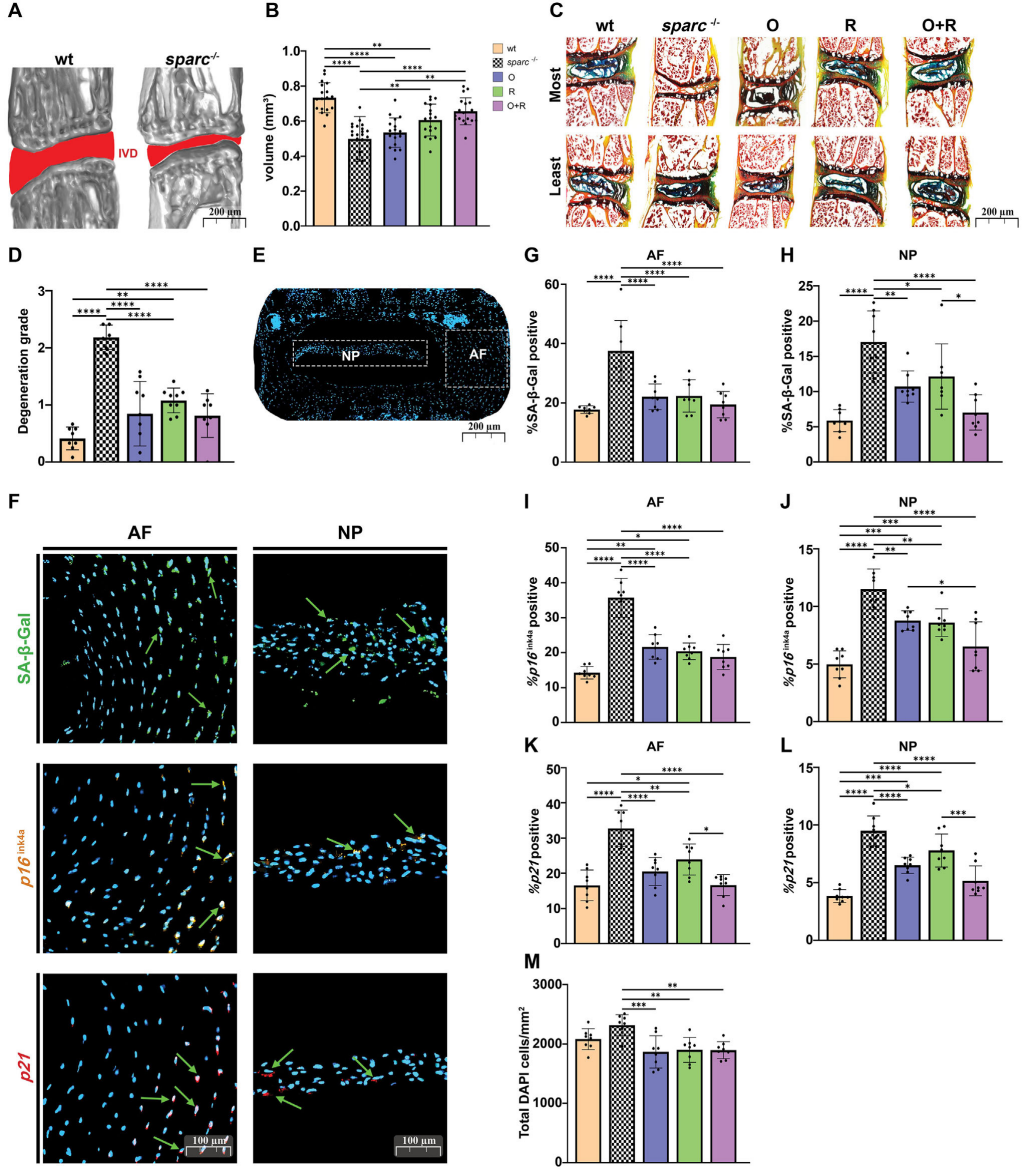
Senotherapeutic treatment increased disc volume, improved IVD integrity, and reduced SnC burden. (A) Representative micro‐CT images outlining the intervertebral discs (IVDs) (red) in 9‐month‐old wt and *sparc* ^−/−^ mice. (B) Quantification of IVD volume (*n* = 6–7 animals (3 IVDs/animal), 3–4 males and 3 females). (C) Representative histological images of lumbar IVDs showing the least and most degenerated discs within each group. (D) Histological degeneration score (*n* = 8–9 animals (3 IVDs/animal); 4–5 males and 4 females). (E) Representative IVD section indicating the annulus fibrosus (AF) and nucleus pulposus (NP) regions (dashed outlines). (F) Representative immunofluorescence (IF) staining of SA‐β‐Gal, *p16^Ink4a^* and *p21* in the AF and NP. Quantification of (G‐H) SA‐β‐Gal‐ positive cells in the AF and NP regions, (I,J) *p16^Ink4a^*‐ positive cells in the AF and NP, and (K,L) *p21*‐positive cells in the AF and NP regions, expressed as a percentage of total cells (DAPI). (M) Quantification of total cells (DAPI) per mm^2^ (*n* = 8 animals (3 IVDs/animal); 4 males and 4 females). Scale bars represent 200 µm in panels A, C, and E and 100 µm in panel F. Statistical comparisons were calculated using an ordinary one‐way ANOVA, with a Tukey's post hoc analysis. Data are presented as means ± SD. *p < 0.05, **p < 0.01, ***p < 0.001, and ****p < 0.0001.

Furthermore, we evaluated whether preventive senolytic treatment could preserve disc volume and IVD health. Previous results where animals with well‐established degeneration were treated showed that the combination treatment was significantly better than single drugs [53]. Here we confirmed a significantly lower lumbar IVD volume in *sparc^−/−^* compared to wt mice (Figure 4A,B and Figure S3A). Treatment with the drugs individually resulted in a slightly preserved disc volume; however, these changes reached statistical significance only following RG‐7112 treatment. Importantly, the combination therapy improved the outcome and preserved disc volume to a higher and more significant level, reaching values comparable to those of wt mice (Figure 4A,B and Figure S3A).

The extent of IVD degeneration was assessed through histological grading using a validated scoring system [53, 54]. Treatment with either o‐vanillin or RG‐7112 resulted in a significantly improved IVD degeneration score compared to vehicle‐treated *sparc* ^−/−^. Notably, combination therapy did not yield an added effect. Furthermore, the o‐vanillin and combination treatment groups achieved degeneration scores comparable to those of wt controls (Figure 4C,D).

In addition to assessing IVD degeneration scores, we evaluated the effect of senolytic treatment on the accumulation of SnCs by quantifying the number of SA‐β‐Gal, *p16^Ink4a^*, and *p21*‐positive annulus fibrosus (AF) and nucleus pulposus (NP) cells (Figure 4E, F–L and Figure S3C–E). SA‐β‐Gal staining was performed as a generalized senescence‐associated readout, while *p16^Ink4a^* and *p21* were used to reflect different stages of senescence; *p21* represents an early marker, while *p16^Ink4a^* represents a late and more stable marker of senescence [67, 68]. Our previous studies demonstrated that systemic administration of o‐vanillin and RG‐7112 reduced *p16^Ink4a^* expression in the IVDs of *sparc^−/−^* mice with established degeneration, supporting their ability to clear SnCs during late‐stage disease [53]. *P21* expression has not previously been examined in this context. All three markers were significantly elevated in the AF and NP regions of *sparc^−/−^* mice compared to wt mice (Figure 4F–L and Figure S3C–E). Treatment with either o‐vanillin or RG‐7112 alone significantly reduced the number of SA‐β‐Gal, *p16^Ink4a^* and *p21* positive cells in the AF and NP, with combination therapy resulting in the most pronounced reduction, approaching levels observed in wt controls (Figure 4 F–L and Figure S3C–E). Comparing the differences between treatments revealed that RG‐7112 was generally less effective in reducing SA‐β‐Gal and *p21*‐positive cells compared to the other two treatments, while o‐vanillin was slightly less effective in removing *p16^Ink4a−^* positive NP cells. Although statistically significant, these differences represented minor changes in the actual number of cells. Quantification of the total number of cells showed that senolytic‐treated *sparc* ^−/−^ discs exhibited significantly lower cellularity, consistent with depletion of SnC populations rather than a shift in marker proportion (Figure 4M). No difference in the total number of cells was found between the three treatment groups.

### 2.5 Senotherapeutics Altered Drug‐Relevant Molecular Targets in the IVD

**FIGURE 5:**
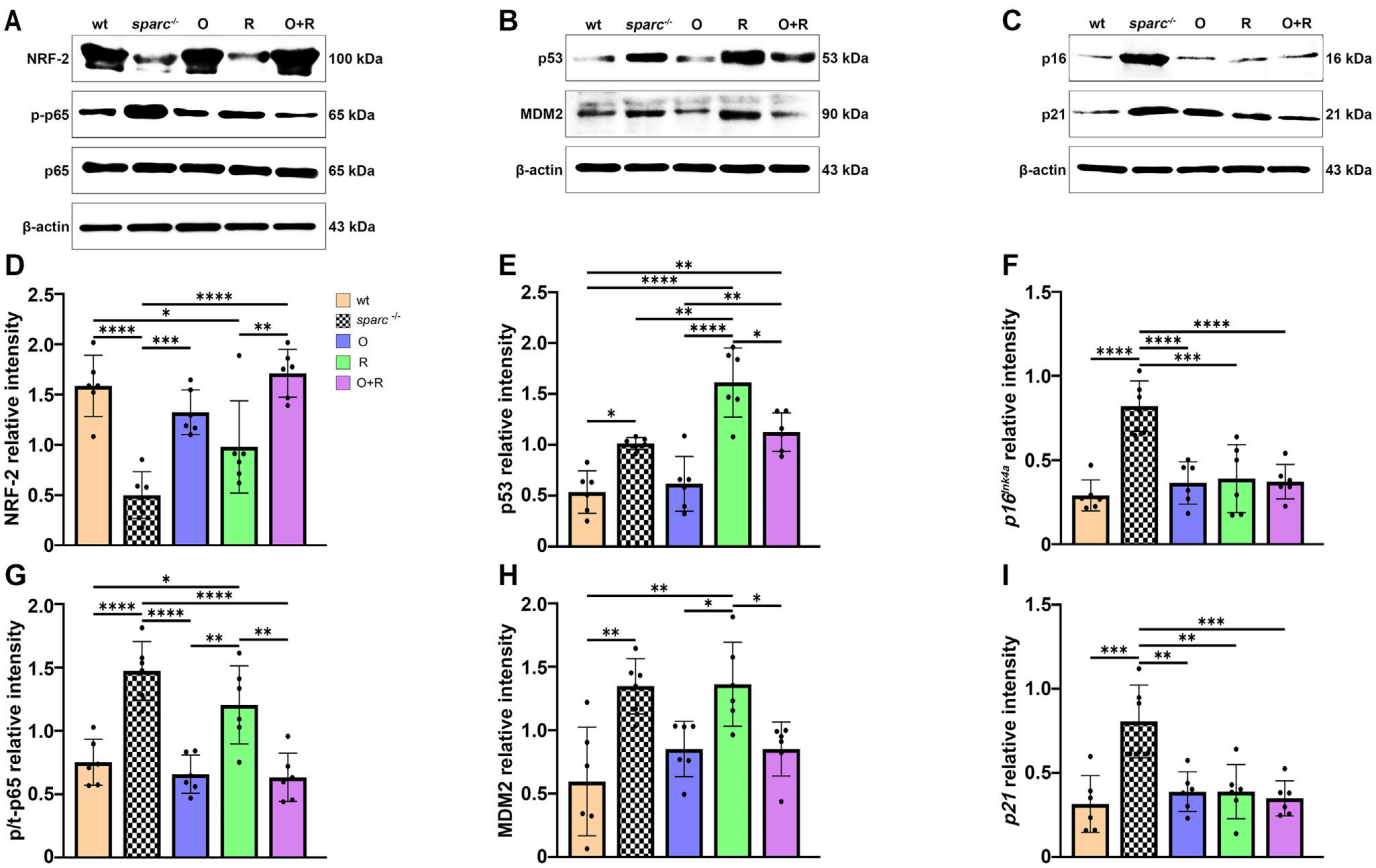
Senotherapeutic treatment altered molecular targets in the IVD. Representative western blot images showing the expression of (A) NRF‐2, p‐p65, p65 and β‐actin, (B) p53, MDM2 and β‐actin, and (C) *p16^Ink4a^*, *p21* and β‐actin in IVD tissue. β‐actin was used as the loading control. Densitometric quantification of (D) NRF‐2, (E) p53, (F) *p16^Ink4a^*, (G) p‐p65/p65, (H) MDM2, and (I) p21 expression normalized to β‐actin (n = 6 animals (3 IVDs/animal); 3 males and 3 females). Data are presented as mean ± SD and were analyzed by ordinary one‐way ANOVA followed by Tukey's post hoc test. *p < 0.05, **p < 0.01, ***p < 0.001, and ****p < 0.0001.

To determine whether the senotherapeutics directly target their expected molecular targets within the IVD, western blot analysis was performed on IVD tissue collected at the termination of the treatment period. Vehicle‐treated *sparc^−/−^* mice exhibited a lower NRF‐2 and higher p‐p65, p53, MDM2, *p16^Ink4a^*, and *p21* expression compared with wt controls (Figure 5A–I). O‐vanillin treatment significantly increased NRF‐2 expression and significantly reduced p‐p65 levels with no significant difference in p53 and MDM2 compared to vehicle‐treated *sparc^−/−^* mice. RG‐7112 treatment significantly increased p53 expression with no significant difference in NRF‐2, p65 and MDM2 compared to vehicle‐treated *sparc^−/−^* mice. Combination treatment significantly increased NRF‐2 and p53 expression and significantly reduced p‐p65 levels compared to vehicle‐treated *sparc^−^* ^/−^ mice. When comparing the single treatment groups with the combination, RG7112 treatment resulted in significantly lower NRF‐2 and significantly higher p‐65, p53 and MDM2 compared to combination treatment. In addition, o‐vanillin, RG‐7112, or their combination significantly reduced *p16^Ink4a^* and *p21* expression in IVD tissue compared with vehicle‐treated *sparc^−/−^* mice (Figure 5A–I).

### 2.6 Senotherapeutics Improved Health and Reduced Senescence in the Endplates

**FIGURE 6:**
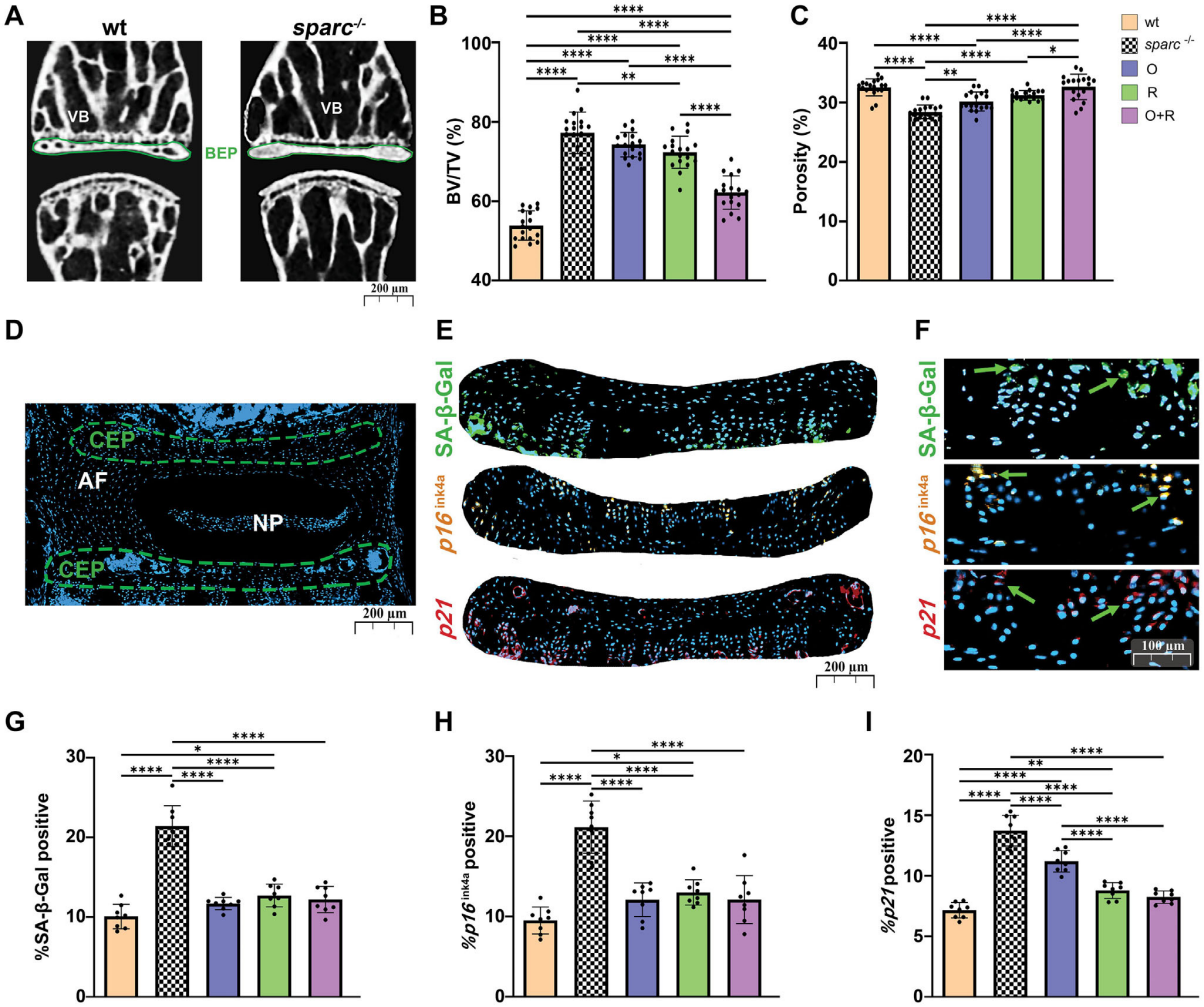
Senolytic treatment enhanced endplate structure and reduced senescent cell accumulation. Representative micro‐CT images of the bony endplate (BEP) outlined in green in wt and *sparc* ^−/−^ mice. (B) Bone volume/total volume (BV/TV) and (C) porosity quantified in the superior BEP at (n = 6 animals per group (3 BEPs/animal); 3 males and 3 females). (D) Representative IVD section illustrating the annulus fibrosus (AF), nucleus pulposus (NP), and cartilage endplates (CEPs) outlined by green dashed lines. (E) Representative immunofluorescence images of SA‐β‐Gal, *p16^Ink4a^* and *p21* staining in the CEP. (F) Higher‐magnification images of SA‐β‐Gal, *p16^Ink4a^* and *p21* positive cells in the CEP. The green arrowheads indicate positive cells. Quantification of (G) SA‐β‐Gal, (H) *p16^Ink4a^*, and (I) *p21* positive cells in the CEP, expressed as percentage of total DAPI cells. DAPI served as a nuclear counterstain (*n* = 8 animals per group (3 CEPs/animal); 4 males and 4 females). Data are presented as means ± SD and analyzed using one‐way ANOVA followed by Tukey's post hoc test. *p < 0.05, **p < 0.01, and ****p < 0.0001. Scale bars represent 200 µm in panels A, D, and E, and 100 µm in panel F.

Degeneration and senescence within the lumbar IVD have been extensively studied in the *sparc* ^−/−^ model, while the cartilage endplates and bony endplates have previously not been carefully examined. The endplates are critical structures regulating nutrient and metabolite transport between the disc and vertebral body [69, 70]. Disruption of endplate integrity can impair disc homeostasis and exacerbate degeneration. Micro‐computed tomography (micro‐CT) was used to investigate the effects of senolytic therapies on bony endplate health. In vehicle‐treated *sparc* ^−/−^ mice, bone volume over tissue volume (BV/TV) was significantly higher (Figure 6A,B and Figure S4A), while porosity was significantly lower in the lumbar bony endplates of *sparc* ^−/−^ compared to wt mice (Figure 6C). These changes may limit nutrient exchange and contribute to a hostile microenvironment for disc cells, thereby promoting degeneration and cellular senescence. Treatment with single drugs resulted in a lower BV/TV, reaching statistical significance for RG‐7112 compared to vehicle‐treated *sparc* ^−/−^ mice. The combination therapy resulted in the most pronounced and robust reduction in BV/TV, significantly lower than single‐drug treatments; however, BV/TV values did not fully reach wt levels. Additionally, both single‐drug treatments showed increased porosity, with the combination therapy resulting in the greatest increase, preserving structural integrity to levels comparable to wt mice (Figure 6C).

To evaluate the effects of senolytic treatment in the cartilage endplates, the number of SA‐β‐Gal, *p16^Ink4a^* and *p21*‐positive cells was quantified (Figure 6D–F). All markers were significantly elevated in *sparc* ^−/−^ compared to wt mice (Figure 6G–I and Figure S4B–D). SA‐β‐Gal positivity was significantly reduced by o‐vanillin and RG‐7112 treatment compared with vehicle‐treated *sparc* ^−/−^ mice. Treatment with either o‐vanillin or RG‐7112 alone led to a lower number of *p16^Ink4a^* and *p21*‐positive cells within the cartilage endplates. Combination treatment also resulted in a significant reduction in SA‐β‐Gal and *p16^Ink4a^* expression relative to *sparc* ^−/−^ mice, though not significantly different from either single treatment. In contrast, *p21* expression was significantly lower following combination treatment compared to both single treatments and approached wt levels.

### 2.7 Senotherapeutics Improved Vertebral Bone Parameters, Reduced Senescence, and Modulated Bone Remodeling

**FIGURE 7:**
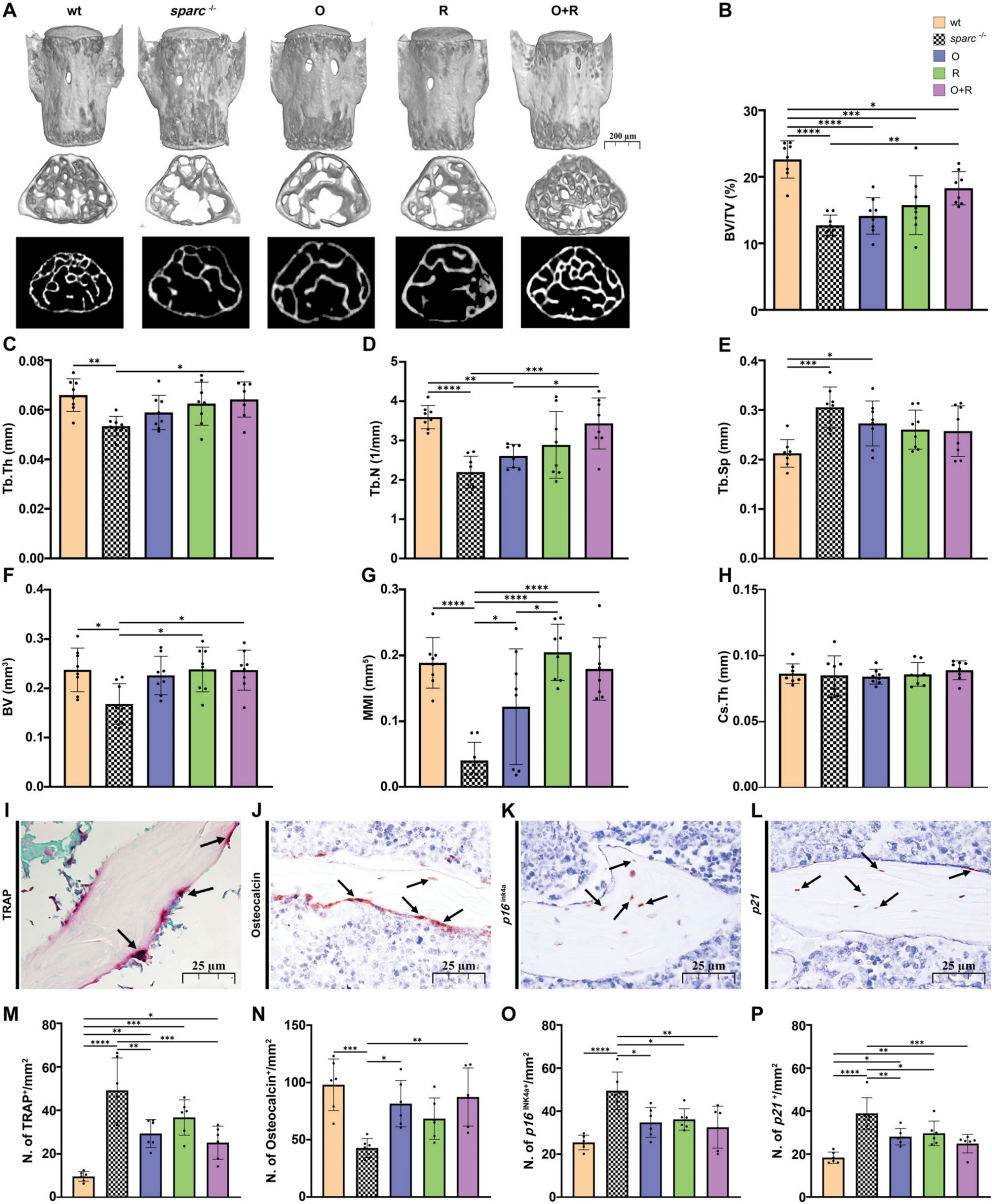
Senotherapeutics improved vertebral bone microarchitecture, decreased senescent cell burden, and modulated bone remodeling. Representative micro‐CT images of (A) vertebral bodies in all groups. Quantification of trabecular bone parameters, including (B) Bone volume/total volume BV/TV, (C) trabecular thickness (Tb. Th), (D) trabecular number (Tb. N), and (E) trabecular separation (Tb. Sp). Quantification of cortical bone parameters, including (F) bone volume (BV), (G) mean moment of inertia (MMI), and (H) cortical thickness (Cs. Th) (n = 8 animals per group(3VBs/animal); 4 males and 4 females). Representative images of (I) TRAP staining, (J) osteocalcin immunohistochemistry, and immunohistochemical staining for (K) p*16^Ink4a^* and (L) *p21* in vertebral bone. Arrows indicate positive cells. Quantification of (M) TRAP‐positive cells, (N) osteocalcin‐positive cells, (O) *p16^Ink4a^*‐positive cells, and (P) *p21*‐positive cells, expressed as number of positive cells/mm^2^, (n = 6 animals per group (3VBs/animal); 3 males and 3 females). Data are presented as mean ± SD and were analyzed by ordinary one‐way ANOVA followed by Tukey's post hoc test. *p < 0.05, **p < 0.01, ***p < 0.001, and ****p < 0.0001. Scale bars represent 200 µm in panel A and 25 µm in panels I‐L.

In addition to IVD degeneration and back pain, *sparc* ^−/−^ mice are known to have osteopenia [71, 72]. As expected, *sparc* ^−/−^ mice displayed significantly lower bone quality compared to the wt group (Figure 7A). The analysis revealed markedly reduced BV/TV (Figure 7B), trabecular thickness (Tb. Th) (Figure 7C), and trabecular number (Tb. N) (Figure 7D), accompanied by a significantly increased trabecular separation (Tb. Sp) (Figure 7E) in *sparc* ^−/−^ mice compared to wt mice. Single‐drug treatment with RG‐7112 significantly improved Tb. Th (Figure 7C). Combination treatment resulted in a robust and significant improvement in all parameters except Tb. Sp (Figure 7B–E). Analysis of cortical bone parameters showed significantly lower bone volume (BV) (Figure 7F) and mean moment of inertia (MMI) (Figure 7G) in *sparc* ^−/−^ compared to wt mice. Single‐drug and combination treatment significantly increased BV (Figure 7F). Improvements in the MMI were observed following single‐drug treatments, and the combination therapy did not provide an additive effect (Figure 7G). The cortical thickness (Cs. Th) parameter did not show a significant difference between the groups (Figure 7H). These findings demonstrate that oral administration of senolytic drugs, particularly in combination, prevents the early deterioration of bone quality in *sparc* ^−/−^ mice.

Vertebral bone remodeling was further evaluated by tartrate‐resistant acid phosphatase (TRAP) staining, which identifies osteoclast activity, and osteocalcin immunohistochemistry, which reflects osteoblastic bone formation (Figure 7I,J,M,N and Figure S5A‐B). Compared with wt controls, vehicle‐treated *sparc* ^−/−^ mice showed a significantly higher number of TRAP‐positive cells and a significantly lower number of osteocalcin‐positive cells in the vertebral bone (Figure 7M,N). O‐vanillin treatment significantly reduced TRAP‐positive cells and significantly increased osteocalcin‐positive cells relative to vehicle‐treated *sparc* ^−/−^ mice. RG‐7112 treatment also reduced TRAP‐positive cells, while the decrease in TRAP and increase in osteocalcin‐positive cells did not reach statistical significance. Combination treatment significantly reduced the number of TRAP‐positive cells and significantly increased osteocalcin‐positive cells compared with vehicle‐treated *sparc* ^−/−^ mice (Figure 7I,J,M,N and Figure S5A,B). No significant differences were detected between the single‐treatment groups and the combination‐treatment group.

To evaluate the effect of senolytic treatment on senescence markers in the vertebral bone, Immunohistochemistry (IHC) staining was performed for *p16* ^Ink4a^ and *p21*‐positive SnCs (Figure 7K, L and Figure S5C,D). Both markers were significantly higher in the vertebral bone of *sparc* ^−/−^ compared to wt mice. Treatment with single senolytic drugs led to a substantial reduction in the number of both *p16^Ink4a^* and *p21 positive* cells in treated *sparc* ^−/−^ mice. Although combination treatment also reduced both markers compared with vehicle‐treated *sparc^−/−^* mice, it did not confer an additional effect beyond that observed with either single treatment (Figure 7O,P). No significant differences were detected among the three treatment groups.

## 3 Discussion

Cellular senescence is increasingly recognized as a causal driver of musculoskeletal degeneration and chronic pain [3]. Within the IVD, accumulation of SnCs promote extracellular matrix breakdown, inflammatory signaling, and discogenic pain [42, 44, 73, 74]. Senolytic strategies that modulate SASP production or selectively eliminate SnCs have shown therapeutic promise in age‐related tissue dysfunction [75, 76]. Since IVD degeneration and accumulation of SnCs are closely linked to back pain, senolytic therapy offers a compelling opportunity for disease prevention [52, 53, 77, 78]. Here, we demonstrate that systemic oral administration of o‐vanillin and RG‐7112 in *sparc^−/−^* mice reduces pain‐related behaviors, suppresses SASP activity, and reduces senescence markers across multiple spinal compartments. Treatment preserved disc volume, maintained vertebral and bony endplates microarchitecture, and reduced senescent astrocytes, microglia, and neurons in the dorsal horn, supporting the potential of senolytics to delay degenerative and pain‐related cascades.

The senolytic properties of o‐vanillin, a natural small molecule, and RG‐7112, a synthetic MDM2 inhibitor were previously established [49, 50, 51, 52, 53, 79]. Both agents selectively cleared SnCs and reduced SASP factors in senescent IVD cells, ex vivo human IVD tissue, and middle‐aged *sparc^−/−^* mice with advanced IVD degeneration [49, 52, 53]. Given the heterogeneous phenotypes and survival mechanisms of SnCs, recent evidence suggests that combining senolytics with distinct molecular targets may enhance therapeutic outcomes [80, 81, 82]. O‐vanillin and RG‐7112 engage different pathways associated with SnC survival. O‐vanillin primarily modulates NF‐κB and NRF‐2–related signaling [49, 83] while RG‐7112 activates p53‐dependent apoptosis through MDM2 inhibition [51, 52]. Our prior findings support that their combined use results in a synergistic effect, producing a more robust reduction in SnC burden and SASP expression across both in vitro and in vivo models of IVD degeneration when the degenerative milieu has already been established [51, 53].

*Sparc^−/−^* mice spontaneously develop IVD degeneration in the lumbar region and pain by 4 months of age [55]. Prior studies in this model have shown that pain‐related behavior responds to analgesic treatment. Morphine alone or in combination with clonidine reverses pain‐like behavior, supporting nociception‐related rather than nonspecific motor or performance changes [54, 55, 56, 62, 84, 85, 86]. Moreover, *sparc^−/−^* mice do not display evidence of systemic inflammation, arguing against a generalized inflammatory state as the primary driver of the observed pain behavior [53].

Initiating senolytic treatment before the onset prevented the emergence of pain‐like behavior, with treated animals exhibiting sensitivity comparable to wt controls at 9 months. Both o‐vanillin and RG‐7112 provided measurable benefits, but combination treatment produced the most robust effects across behavioral assays. Prior studies have shown that SnC clearance mitigates cartilage and disc degeneration and reduces pain in osteoarthritis models [87, 88, 89]. Our data extends these findings by demonstrating that early senotherapy interrupts the trajectory toward chronic pain, establishing senescence as a mechanistic link between degeneration and nociception.

Neuroinflammation is a critical component of chronic pain pathogenesis. Elevated *p16^Ink4a^* expression was observed in the dorsal horn of *sparc^−/−^* mice, consistent with prior reports implicating spinal senescence in pain states [53, 58, 90, 91]. Senescent neurons, glia, and astrocytes accumulate in pain models and patient tissues [92, 93, 94, 95, 96, 97, 98, 99, 100, 101], and their SASP promotes neuroinflammation and sensitization [58, 90, 96]. Senolytic interventions in spinal cord injury reduce inflammation, glial scarring, and promote functional recovery [102]. Consistently, senescent astrocytes, microglia, and neurons were identified in the dorsal horn, and were all reduced by senotherapy. Unlike peripheral tissues, combination treatment did not show additive effects in the spinal cord, suggesting that CNS‐resident SnCs may share common vulnerabilities. These findings are consistent with the role of senescent neural and glial populations in central sensitization and support their removal as a potential strategy to restore spinal homeostasis and alleviate pain [58, 90, 96].

Senolytic treatment broadly suppressed SASP activity within IVDs. The lumbar IVDs of vehicle‐treated *sparc^−/−^* animals displayed elevated levels of inflammatory cytokines and chemokines with o‐vanillin and RG‐7112 treatment reducing all 15 factors measured. In contrast, interventions in middle‐aged *sparc^−/−^* animals attenuated only 10 factors [53], suggesting that early therapy intercepts pro‐inflammatory feedback before stabilization. Notably, chemokines such as CXCL‐1, CXCL‐5, and CXCL‐10, all of which have been linked to ECM degradation and senescence propagation [52, 103, 104, 105], were strongly reduced. Similarly, CCL‐2 and CCL‐7, mediators of immune recruitment and joint inflammation [52, 53, 105], were suppressed. Key cytokines central to disc degeneration and osteoarthritis, including IL‐1β, TNF‐α, and IL‐6, were also decreased, with IL‐6 showing an additive reduction under combination treatment. Downregulation of VEGF‐α and RANKL further indicates that early senotherapy limits angiogenic and osteoclastogenic cascades [106]. Collectively, these results demonstrate that early intervention achieves more comprehensive inflammatory suppression than late treatment.

Histological analyses confirmed reductions in SA‐β‐Gal, *p16^Ink4a^* and *p21*‐positive cells in IVDs, cartilage endplates, and vertebral bone. Notably, clearance of *p21*‐positive cartilage endplate cells required combination treatment, suggesting differential vulnerability among senescent subpopulations. This aligns with evidence that *p21*‐driven senescence can resist single‐agent therapy [107, 108, 109, 110]. Prior studies demonstrate enhanced efficacy of combining senolytics [45, 77, 111, 112, 113, 114, 115, 116, 117], and our findings reinforce this in disc and bone tissues. Studies using a combination of dasatinib and quercetin, similar to o‐vanillin and RG‐7112, ameliorated IVD degeneration and reduced senescence and SASP in aged wildtype and young poor‐healer SM/J mice [118, 119, 120].

Our oral treatment contrasts previous intraperitoneal regimens [77] and may offer greater translational relevance for preventive therapeutic strategies. Although localized intradiscal delivery or the use of a more disc‐restricted degeneration model would provide tissue‐specific mechanistic attribution, oral administration provides a clinically more practical and less invasive approach for long‐term intervention. We intentionally selected a model characterized by progressive extracellular matrix degeneration, reflecting the gradual course of human spine degeneration associated with back pain, rather than more acute injury established with models such as needle‐ or stab‐induced disc disruption.

It is well established that the NP and the inner two‐thirds of the AF lack direct vascular supply and instead rely on diffusion from capillaries located in the subchondral region of the endplate for nutrients [121, 122]. With advancing age, calcification of the endplates may ultimately compromise nutrient delivery to the IVD [88, 123, 124, 125]. In the present study, *sparc^−/−^* mice exhibited more calcification and less porosity of the lumbar endplates than wt animals, a pattern consistent with human disc degeneration [126]. Treatment with o‐vanillin and RG‐7112 reduced bone volume and increased porosity of the endplates. These findings suggest that senolytic intervention may partially reverse degenerative endplate changes and improve the structural conditions that support solute transport between the vertebral body and the IVD.

Targeting SnCs in the bony endplates with senolytic therapy may help restore this nutrient pathway and support disc health. Prior studies in aged mice have reported reductions in IVD height associated with vertebral bone loss and marked disruption of endplate vasculature, changes that likely impair nutrient transport [123]. Additionally, recent findings indicate that osteoclasts within the vertebral endplate undergo senescence in response to injury and aging, with elevated levels of TRAP, SA‐β‐Gal, and *p16^Ink4a^* observed in both aged and mechanically injured models [88]. In our study, treatment with o‐vanillin and RG‐7112 significantly reduced the number of SA‐β‐Gal, *p16^Ink4a,^* and *p21*‐positive cells in the cartilage endplates and increased the porosity of the bony endplates.

Our prior pharmacokinetic analyses showed that both compounds are detectable in IVD tissue and spinal cord following oral administration [53], supporting direct effects within the IVD. In the present study, western blot analysis of IVD tissue supports local target effects following oral administration, with o‐vanillin modulating NRF‐2/NF‐κB‐related signaling and RG‐7112 affecting the p53/MDM2 pathway within the disc. These findings are consistent with the known mechanisms of the compounds and strengthen the interpretation that the observed reduction in senescence markers and preservation of disc structure is linked, at least in part, to direct biological activity within IVD tissue. This interpretation is further supported by our previous ex vivo studies in degenerating intact human and mouse discs, in which each senolytic treatment reduced SnC burden and SASP expression while improving disc matrix homeostasis [52, 53, 85].

*Sparc^−/−^* mice display osteopenia, which is thought to contribute to the onset and progression of back pain [71, 127]. Treated animals showed preserved vertebral trabecular and cortical architecture with reduced senescent osteocytes and osteoclasts, consistent with prior senolytic studies in skeletal aging [112, 115, 123, 128, 129, 130]. Importantly, vertebral bone remodeling was altered in treated animals, osteoclast‐associated activity was reduced, and osteoblast activity was enhanced following senolytic treatment. These findings suggest that o‐vanillin and RG‐7112 help rebalance vertebral bone turnover toward a less catabolic state. Unlike Navitoclax, which may impair osteoblast differentiation [130], or dasatinib and quercetin, which showed variable effects in vertebrae [77], *o*‐vanillin and RG‐7112 preserved bone integrity, highlighting distinct therapeutic advantages.

Mechanistically, o‐vanillin and RG‐7112 target divergent survival pathways. RG‐7112 induces apoptosis in p53/*p21*‐driven SnCs, whereas o‐vanillin modulates NF‐κB and *p16^Ink4a^*‐driven populations [49, 52, 53]. Additive suppression of IL‐6 and enhanced clearance of *p21*‐positive cells support complementary activity. Importantly, treatment timing strongly influenced outcomes. In middle‐aged *sparc^−/−^* mice, both agents were required at full dose, and only combination treatment achieved comprehensive effects [53]. In contrast, early intervention allowed effective suppression with monotherapy, indicating greater vulnerability of nascent SnCs.

In summary, the *sparc* ^−/−^ mouse model exhibits alterations in multiple musculoskeletal tissues implicated in back pain. Our data establishes that oral senotherapy with o‐vanillin and RG‐7112 likely reflects combined actions across spinal tissues. It prevents SnC accumulation, suppresses SASP production, preserves disc and bone integrity, and prevents chronic pain behaviors in *sparc^−/−^* mice. These findings identify cellular senescence as a mechanistic driver of spine degeneration and pain and demonstrate that early intervention provides durable protection across multiple spinal compartments. The results support translation of senotherapeutic strategies toward preventive clinical applications in individuals at risk for early‐onset or genetically driven disc degeneration [131].

## 4 Materials and Methods

### 4.1 Study Design

This study evaluated the preventive effects of the senolytic compounds o‐vanillin and RG‐7112 on IVD degeneration and associated back pain. The *sparc* ^−/−^ mouse model, which we have previously demonstrated to exhibit early‐onset of IVD degeneration, back pain, and increased accumulation of SnCs in the spine was employed [53]. Based on this phenotype, early senolytic treatment was assessed for its ability to prevent or reduce the accumulation of SnCs and preserve spine health. To further characterize the effect of senolytic treatment on pain modulation, pain behavior was assessed monthly using grip strength, von Frey, and acetone‐evoked tests. We also performed immunostaining for markers of activated astrocytes, microglia, and neurons across spinal tissues from untreated wt mice and both treated and untreated *sparc* ^−/−^ mice. At the endpoint, lumbar IVDs were harvested for SASP factor analysis by Luminex multiplex assay and Western blot analysis. SnC depletion was evaluated by SA‐β‐Gal, *p16^Ink4a^* and *p21* immunofluorescence (IF) in IVDs, cartilage endplates, vertebral bone, and spinal cord. Vertebral bone remodeling was further assessed by TRAP staining and osteocalcin immunohistochemistry. Structural improvements in discs and bone were assessed by micro‐CT for disc volume, endplate porosity, and bone microarchitecture, and by FAST histological grading for IVD degeneration.

### 4.2 Animals and Housing

All animal procedures were approved by the McGill University Animal Care Committee in accordance with the Canadian Council on Animal Care guidelines (Protocol number: MUHC‐ 10007). Age‐matched male and female C57BL/6N wild‐type and *sparc* ^−/−^ mice were used across all experimental groups. The *sparc* ^−/−^ mouse model, originally generated on a C57BL/6×129SVJ background, was maintained through successive backcrossing to the C57BL/6N strain [56, 132].

Mice were housed in groups of two to four per cage in ventilated polycarbonate cages (Allentown) under standard conditions (12‐hour light/dark cycle, controlled temperature, and humidity). Environmental enrichment was provided via cotton nesting material, and cages were lined with corncob bedding (Envigo). Animals had ad libitum access to irradiated, soy‐free extruded rodent chow and filtered water.

Group sizes were determined based on prior data and power calculations from previous experiments involving *sparc* ^−/−^ mice, ensuring sufficient statistical sensitivity to detect both genotype and treatment‐related effects (Table S2) [53, 62, 86, 133, 134, 135, 136]. All analyses were derived from a single treatment cohort. All animals underwent behavioural testing. At the study endpoint, animals from this cohort were allocated across downstream assays according to tissue requirements and assay compatibility. Disc levels used for all downstream analyses (L2–L5) were selected based on our previous findings showing that SnC burden and degeneration severity are greater in the lower lumbar region of *sparc* ^−/−^ mice [53]. Accordingly, disc selection was not random; all analyses were performed on L2‐L5 discs, based on anatomical and disease‐relevance criteria established in the model.

A detailed breakdown of assay allocation, spinal levels analyzed, sample size, and sex distribution for each experimental outcome is provided in Supplementary Table S3.

### 4.3 Randomization and Blinding

Animals were randomly assigned to treatment groups. Investigators remained blinded to genotype and treatment allocation throughout the study. This blinding was maintained during tissue processing, immunostaining, microscopic image acquisition, ROI selection, histological grading, and all quantitative analyses. Cell counting, including quantification of total DAPI‐positive nuclei and SA‐β‐Gal, *p16^Ink4a^* and *p21*‐positive cells, was performed independently by three blinded evaluators. Histological scoring was likewise conducted independently by three blinded evaluators. Statistical analyses were performed using datasets, and group identities were disclosed only after all measurements and analyses had been completed.

### 4.4 Treatment Regime and Endpoints

Four‐month‐old male and female mice were randomly assigned to five treatment groups. Both *sparc* ^−/−^ and wt mice received oral gavage weekly for a duration of 5 months. *Sparc* ^−/−^ mice were treated with one of the following senolytics: o‐vanillin (O; 100 mg/kg), RG‐7112 (R; 5 mg/kg), a combination of both drugs (O+R; o‐vanillin + RG‐7112), or vehicle control (0.01% dimethyl sulfoxide in saline). Wt mice received only vehicle treatment to serve as baseline controls.

The frequency and dosage of drug treatment, as well as the experimental endpoints, were determined based on previous studies [53, 62, 86, 133, 134, 135, 136]. The initiation of treatment was chosen based on the onset of the pain phenotype and IVD degeneration. *Sparc* ^−/−^ mice begin to exhibit signs of back pain and lumbar disc degeneration at 4 months of age [54, 55, 56]. Our objective was to assess whether senolytic treatment, with o‐vanillin and RG‐7112, administered either as a single or in combination, could provide a protective effect against the progression of these phenotypes.

### 4.5 Pain Behavior

Pain‐related behavior was evaluated as previously described [53, 55, 56, 62, 84, 85]. All behavioral testing was conducted in a dedicated room under standard indoor lighting between 8:00 a.m. and 12:00 p.m. To reduce stress, mice were habituated to the testing environment for 1 h, followed by an additional 1‐hour habituation period in Plexiglas testing boxes placed on a metal mesh platform when applicable. Each behavioral test lasts between 30 s and 5 min per animal. Behavioral testing was performed over two consecutive days: grip strength on the first day, and von Frey and acetone tests on the second. This schedule was maintained throughout the study, including baseline measurements. Animals were closely monitored for signs of distress or discomfort during testing before being returned to the animal facility.

#### 4.5.1 Grip Strength

Axial discomfort was assessed using a grip strength meter (Stoelting Co.). Mice were encouraged to grasp the bar with their forepaws while gentle, steady traction was applied via the tail until they released their grip. The maximal force was recorded in grams [136]. For each session, grip strength was measured three times per mouse, and the values were averaged to obtain a final score. To avoid fatigue and stress, animals were returned to their home cages for approximately 15 min between measurements.

#### 4.5.2 Acetone‐Evoked Behavior Test for Cold Sensitivity

Behavioral reaction to a cold stimulus was used to determine radiating pain. Acetone (30 to 50 µL) was applied to the left and right hind paws, and the total duration of nocifensive behavior (paw lifting, shaking, and scratching) was recorded for 30 s [136].

#### 4.5.3 Von Frey Test for Mechanical Sensitivity

Mechanical sensitivity was assessed using von Frey filaments (Stoelting Co.) applied to the plantar surface of the left and right hind paws. Each filament was applied with enough force to slightly bend the filament and held in place until a withdrawal response occurred or for a maximum of 5 s, whichever came first [53, 56]. Stimulus intensity ranged from 0.6 to 4.0 g, corresponding to filament numbers 3.22, 3.61, 3.84, 4.08, and 4.17. The 50% paw withdrawal threshold was calculated using the up‐down method [137], providing a quantitative measure of mechanical hypersensitivity.

### 4.6 Luminex Multiplex Assay

SASP factor release from the IVDs was evaluated at the termination of treatment. Animals were deeply anesthetized via intraperitoneal injection of a mixture containing ketamine (100 mg/kg), xylazine (10 mg/kg), and acepromazine (3 mg/kg). Lumbar IVDs (L2–L5) with intact cartilage endplates, without vertebral bone, were dissected for analysis. To assess the local secretory phenotype of the disc tissue under standardized conditions, IVDs were maintained for 48 h in serum‐free DMEM supplemented with 1×GlutaMAX, penicillin (10 U/mL), and streptomycin (10 µg/mL) in an incubator [53]. The DMEM was then collected for protein analysis. This approach was used to measure SASP‐factor release from the discs. The concentration of 15 proteins (CXCL‐1, CXCL‐5, CXCL‐9, CXCL‐10, CCL‐2, CCL‐7, IL‐1β, IL‐2, IL‐6, IL‐10, TNF‐α, IFN‐γ, VEGF‐α, RANKL, and M‐CSF) were measured using a Luminex multiplex assay (PPX‐15‐MXGZF4V, ThermoFisher Scientific, Toronto, ON, Canada), according to the manufacturer's instructions. Data acquisition was performed using the MAGPIX system with xPONENT software, and results were processed and analyzed using the ProcartaPlex Analysis App. Cytokine concentrations were normalized to the total protein content of each sample and are presented as pg/mg total protein.

### 4.7 Western Blot Analysis

Western blot analysis was performed on IVD tissue extracted in 4 M guanidine hydrochloride (GuHCl, pH 5.8) [138, 139, 140]. The extracted proteins were collected using ethanol precipitation. The precipitated proteins were resuspended in nuclease‐free water, and total protein was quantified using the Pierce BCA Protein Assay Kit (23225, ThermoFisher Scientific, Toronto, ON, Canada) as per the manufacturer's guidelines and a TECAN Infinite M200 PRO plate reader equipped with i‐control 1.9 Magellan software (TECAN, Männedorf, Switzerland). Proteins were separated on 1.0 mm Novex 4–20% Tris‐Glycine Mini Protein Gels using the NuPAGE electrophoresis system under reducing conditions. Proteins were transferred onto nitrocellulose, the membranes were blocked in 5% milk in TBST, and exposed to primary and secondary antibodies (Supplementary Table S4). Immunoreactive bands were detected using enhanced chemiluminescence (ECL) and imaged with the ImageQuant LAS 4000 system. Band intensities were quantified by densitometric analysis using ImageJ. Protein levels were normalized to β‐actin, and phospho‐p65 expression was further normalized to total p65.

### 4.8 Histological Analysis

#### 4.8.1 Sample Preparation

Animals were deeply anesthetized, and transcardial perfusion was performed using a vascular rinse, and tissue fixation by 4% paraformaldehyde (PFA) in 0.1 M phosphate buffer (PBS) (pH 7.4) at room temperature. The T13–S1 spinal segment was harvested and post‐fixed overnight in 4% PFA at 4°C. Samples were then decalcified in 4% ethylenediaminetetraacetic acid (EDTA) in PBS at 4°C for 2–3 weeks with regular solution replacement. The decalcification endpoint was determined manually by assessing residual resistance of the vertebral bone to needle penetration, and only fully decalcified samples were processed further [141]. After decalcification, tissues were cryoprotected in 30% sucrose in PBS for 4 days at 4°C, then embedded in optimal cutting temperature (OCT) compound (Tissue‐Tek, Sakura Finetek, Torrance, CA, USA). Sagittal sections were cut at 16 µm thickness using a cryostat (Leica CM3050S, Leica Microsystems Inc., Concord, Ontario, Canada), thaw‐mounted onto gelatin‐coated glass slides, and stored at −20°C until further processing.

#### 4.8.2 FAST Staining

Staining was performed on sagittal spinal sections using the FAST protocol, as previously described by Millecamps et al. [53, 54, 55, 56, 84, 85, 86]. IVD degeneration severity grading scale: Grade 0: Healthy IVDs display intact structure, a clear distinction between outer AF and inner NP and negatively charged proteoglycans; grade 1: The changes in extracellular components and IVD integrity were identified as grade 0, normal structure, but the loss of proteoglycans in inner NP; grade 2: internal disruption (loss of boundary) between NP and AF; grade 3: bulging of NP in dorsal aspect; and grade 4: herniation. Each value represents the average grading score of 3 IVDs (L2‐L5) per animal [53, 55].

### 4.9 Immunofluorescence Staining

Sagittal spinal sections were processed for IF detection of senescence markers following the protocol previously described by Mannarino et al. [53]. Additionally, three spinal cord sections per animal were randomly selected, spanning the lumbar spinal cord for each antibody. Sections were incubated in blocking buffer for 1 h at room temperature. Slides were then incubated with appropriate antibodies (Table S4) in blocking buffer overnight at 4°C. After PBS washes, sections were incubated for 1.5 h at room temperature with appropriate secondary antibodies (Table S4) in blocking buffer. DAPI (1:50,000 in PBS; Sigma‐Aldrich, Oakville, ON, Canada) was briefly applied, and slides were washed another three times for 5 min. Coverslips were mounted using Aqua‐Poly/Mount (Polysciences Inc., Warrington, PA, USA). Fluorescence images were captured at 10× magnification using an Olympus BX51 microscope equipped with an Olympus DP71 camera. For the spinal cord, colocalization of *p16^Ink4a^* with GFAP, Iba1, and NeuN in the dorsal horn was quantified using ImageJ. The number of *p16^Ink4a^*‐positive cells was expressed as the number of double‐labelled cells per mm^2^ relative to each marker (GFAP, Iba1 and NeuN). For the IVD and endplate regions, the percentage of *p16^Ink4a^* or *p21*‐positive cells was calculated by dividing the number of positively stained nuclei by the total number of DAPI‐stained nuclei. Approximately 5,000‐7000 cells were quantified per treatment group.

#### 4.9.1 TRAP Staining

TRAP staining was performed on sagittal spinal sections using the TRAP/ALP Stain Kit (294‐67001, Wako Pure Chemical Industries, Osaka, Japan). Spine sections were rinsed in distilled water before staining. TRAP staining solution was prepared according to the manufacturer's instructions. The reaction was stopped by washing the sections in distilled water, and stained sections were mounted and imaged by light microscopy. TRAP‐positive cells were identified based on the presence of the characteristic reddish‐purple reaction product and quantified in the region of interest.

### 4.10 Immunohistochemistry

Sagittal spinal sections were processed for IHC detection of osteocalcin, *p16^Ink4a^*, and *p21*. The sections were subjected to antigen retrieval and endogenous peroxidase activity blockage before IHC staining and detection [142]. Sections were exposed to primary and secondary antibodies (Table S4) and counterstained with hematoxylin before detection using the Rabbit‐specific horseradish peroxidase (HRP)/3,3‐diaminobenzidine (DAB) Detection IHC Kit (ab64261, Abcam, Cambridge, UK) according to the manufacturer's instructions.

### 4.11 Senescence‐Associated β‐Galactosidase Staining

Senescence‐associated SA‐β‐Gal staining was performed on frozen sections using the CellEvent Senescence Green Detection Kit (C10851, ThermoFisher Scientific, Toronto, ON, Canada), according to the manufacturer's instructions. Sections were mounted in DAPI‐containing mounting medium. Images were acquired using a fluorescence microscope with an Alexa Fluor 488/FITC‐compatible filter set. SA‐β‐Gal ‐positive cells were quantified in the AF, NP, and cartilage endplate regions. Data are presented as the percentage of SA‐β‐Gal ‐positive cells per DAPI cells.

### 4.12 Micro‐CT Analysis

Micro‐CT imaging and analysis were performed as previously described [53, 123, 143]. High‐resolution scans of fixed spines were acquired from the L2 to L5 vertebral levels to evaluate three‐dimensional (3D) structural features. Scans were conducted using a Skyscan 1172 micro‐CT system (Bruker, Kontich, Belgium) with a spatial resolution of 15 µm. The system was equipped with a 0.5‐mm aluminum filter and operated at 45 kV and 220 µA with a 360° rotation. Images were captured at 0.4° rotation steps, averaging four frames per image, with an exposure time of 1.46 s per frame. Image reconstruction was performed using the nRecon software (v1.7.1.0, Bruker), and quantitative analysis was carried out using CTAn software (v1.18.8.0, Bruker). CTVOX (v3.3, Bruker) was used for 3D visualization of bone morphology and disc architecture. Transverse cross‐sectional images were analyzed to assess disc volume and bone structure. Disc volume was measured by manually defining an ROI encompassing the space between adjacent endplates. For trabecular bone analysis, the ROI was delineated between the endplate and transverse process within the vertebral body (Figure S3B). From these 3D datasets, standard trabecular bone parameters were calculated, including bone volume over total volume (BV/TV), trabecular thickness (Tb. Th), trabecular number (Tb. N), and trabecular separation (Tb. Sp). Cortical bone parameters were assessed using two‐dimensional (2D) measurements, including cortical bone volume (BV), cortical thickness (Cs. Th), and polar moment of inertia (MMI).

### 4.13 Statistical Analysis

Power analysis was conducted with an alpha (α) level of 0.05 and a power (1‐β) of 80–90%. Based on these parameters, a sample size of 6 for Luminex assay, endplate micro‐CT, vertebral bone staining and western blot analysis, and 8 to 10 animals for the other experiments per group (wt and *sparc^−/−^*) was determined to be sufficient to detect statistically significant differences, with 10 to 15 animals required if sex‐specific effects were present (Table S2). All statistical analyses were performed using GraphPad Prism 10. Data are presented as mean ± standard deviation (SD), and significance was set at *p* ≤ 0.05. Depending on the experimental design, comparisons were made using repeated‐measures one‐way ANOVA, or two‐way ANOVA, followed by Dunnett's or Tukey's post hoc tests where appropriate.

## Author Contributions

**Conceptualization**: L.H., L.S.S., J.A.O., H.C., and S.G. **Methodology**: L.H., L.S.S., J.A.O., H.C., S.G., and M.Mi. **Investigation**: S.G., H.C., M.Ma., and J.S. **Visualization**: S.G., M.Ma., H.C. and J.S. **Supervision**: L.H., L.S.S., H.C., and S.G. **Writing‐ original draft**: L.H. and S.G. **Writing‐ review and editing**: L.H., H.C., S.G., M.Ma., L.S.S, M.Mi., J.A.O., and J.S.

## Conflicts of Interest

The authors declare no conflicts of interest.

## Supporting information




**Supporting File**: advs75814‐sup‐0001‐SuppMat.docx.

## Data Availability

The data that support the findings of this study are available from the corresponding author upon reasonable request.
