# Supplementary material for: Senolytic Therapy as a Preventive Strategy for Spine Degeneration and Pain
Source: Adv Sci (Weinh). 2026 May 25;13(46):e22770. doi: 10.1002/advs.202522770 (PMC13336069; doi:10.1002/advs.202522770)
Supplement: Supplementary file 1 [file ADVS-13-e22770-s001.docx]

Supporting Information

**Senolytic Therapy as a Preventive Strategy for Spine Degeneration and Pain**

*Saber Ghazizadeh^1,2,6^, Hosni Cherif^1,2^, Matthew Mannarino^1,2,6^, Juiena Sagir^1,2^, Magali Millecamps^5,6,7^, Jean A. Ouellet^1,2,4^, Laura S. Stone^3,6^, Lisbet Haglund^1,2,4,5,6^**

^1^Department of Surgery, Orthopaedic Research Lab, McGill University; Montreal, QC, Canada.

^2^Department of Surgery, McGill Scoliosis and Spine Group, McGill University; Montreal, QC, Canada.

^3^Department of Anesthesiology, University of Minnesota; Minneapolis, Minnesota, USA.

^4^Shriner's Hospital for Children; Montreal, QC, Canada.

^5^ABC-platform (Animal Behavioral Characterization) at the Alan Edwards Centre for Research on Pain; McGill University, Montreal, QC, Canada.

^6^Alan Edwards Centre for Research on Pain (AECRP); McGill University, Montreal, QC, Canada.

^7^Département de biomédecine vétérinaire, Faculté de médecine vétérinaire, Université de Montréal, Saint-Hyacinthe, QC, Canada.

*Corresponding author:

Email: [lisbet.haglund@mcgill.ca](mailto:lisbet.haglund@mcgill.ca);

ORCID: <https://orcid.org/0000-0002-1288-2149>

**Figure S1. Within-group temporal changes in behavioral measures, body weight, and mortality.** Within-group comparisons for **(A)** cold sensitivity measured by acetone-evoked behavior, **(B)** axial discomfort measured by grip strength, and **(C)** mechanical sensitivity measured by von Frey testing across time points and in each group. Longitudinal body weight measurements in **(D)** male and **(E)** female mice across the study period. **(F)** Mortality across the 20-week treatment period in all experimental groups. Data are presented as mean ± SD. Behavioral data were analyzed by two-way ANOVA followed by Tukey’s post hoc test. n = 15 animals per group (8 males and 7 females). **P* < 0.05, ***P* < 0.01, ****P* < 0.001 and *****P* < 0.0001.

 **Figure S2. Representative spinal cord images for *p16^Ink4a^*, GFAP, Iba1, NeuN, and DAPI colocalization.** Representative images from all five experimental groups showing **(A)** *p16^Ink4a^*/DAPI staining, **(B)** *p16^Ink4a^*/GFAP/DAPI staining, **(C)** *p16^Ink4a^*/Iba1/DAPI staining, and **(D)** *p16^Ink4a^*/NeuN/DAPI staining in the dorsal horn of the spinal cord. White dashed lines delineate the dorsal horn region used for quantification. **(E)** Representative images showing negative controls for secondary antibodies Alexa 488 (green), CY3 (orange), Alexa 594 (red), and Alexa 647 (red) used for the staining of p*16^Ink4a^*, GFAP, Iba1, and NeuN, respectively. All sections were counterstained with DAPI. Scale bars represent 100 μm in the combination (O+R) images in panels A–E.

 **Figure S3. Representative images of** micro-CT, **IVD volume and senescence markers in AF and NP. (A)** Representative micro-CT images showing IVD volume (red) in all five experimental groups. **(B)** Representative image of the region of interest (ROI; red dashed box) used for selection of the micro-CT measurement area. The lower region corresponds to the endplate, while the upper region includes the transverse processes, which served as anatomical landmarks for ROI selection. Representative immunofluorescence images of **(C)** SA-β-Gal, **(D)** *p16^Ink4a^*, and **(E)** *p21* staining in AF and NP cells across all experimental groups. DAPI served as a nuclear counterstain. Scale bars, 200μm in panels A and B, and 100μm in panels C, D, and E in the combination (O+R) images.

 **Figure S4. Representative images of the superior bony endplate and senescence markers in the cartilage endplate. (A)** Representative micro-CT images of the five experimental groups showing the bony endplate (BEP), outlined in green, region selected for micro-CT parameters quantification. Representative immunofluorescence images of **(B)** SA-β-Gal, **(C)** *p16^Ink4a^*, and **(D)** *p21* staining in the cartilage endplate (CEP) across all experimental groups. Representative negative control images for **(E)** Alexa 488 (SA-β-Gal), **(F)** CY3 (*p16^Ink4a^*), and **(G)** Alexa 647 (*p21*) in the CEP. DAPI served as a nuclear counterstain. Scale bars, 200μm in panel A, (E-G) and 100μm in panels B, C, and D images.

 **Figure S5. Representative images of vertebral bone markers across experimental groups.** Representative images of vertebral bone sections stained for **(A)** TRAP, **(B)** osteocalcin, **(C)** *p16^Ink4a^*, and **(D)** *p21* in all five groups. For each marker, the lower row shows the 40× magnification of the outlined box in A-D, respectively. Scale bars, 100 μm in the upper rows of panels A-D, 50 μm in the lower rows of panels A-D.

**Supplementary Table 1:** Average concentrations normalized to total protein (pg/mg) of SASP factors collected in serum-free DMEM from L2-L5 IVD in each group. Data is presented as mean ± SD.

| **Groups**  **SASP factors** | **wt** | ***sparc^-/-^*** | **O** | **R** | **O+R** |
| --- | --- | --- | --- | --- | --- |
| **CXCL1** | 13.70± 5.18 | 54.79 ± 14.29 | 25.69 ± 4.20 | 30.46 ± 9.09 | 25.31 ± 6.91 |
| **CXCL5** | 17.28 ± 4.08 | 78.32 ± 12.77 | 36.32 ± 7.58 | 42.23 ± 7.58 | 38.10 ± 9.15 |
| **CXCL9** | 40.80 ± 11.80 | 203.97 ± 48.18 | 88.40 ± 47.37 | 72.68 ± 16.74 | 47.95 ± 10.97 |
| **CXCL10** | 5.20 ± 2.57 | 33.80 ± 6.36 | 9.78 ± 6.15 | 7.55 ± 1.53 | 4.74 ± 1.85 |
| **CCL2** | 271.47 ± 78.73 | 994.29 ± 125.19 | 530.01 ± 178.80 | 631.59 ± 119.30 | 583.04 ± 114.60 |
| **CCL7** | 45.07 ± 12.88 | 142.72 ± 8.26 | 67.18 ± 16.24 | 72.40 ± 5.88 | 69.82 ± 6.75 |
| **IL-1β** | 1.67 ± 0.47 | 8.72 ± 1.29 | 3.59 ± 1.07 | 3.98 ± 0.66 | 3.16 ± 0.64 |
| **IL-2** | 6.95 ± 1.22 | 24.61 ± 2.93 | 10.98 ± 2.21 | 12.13 ± 1.14 | 10.73 ± 1.44 |
| **IL-6** | 676.08 ± 273.40 | 7066.35 ± 842.72 | 4439.60 ± 642.72 | 4745.93 ± 632.91 | 3807.28 ± 834.41 |
| **IL-10** | 9.37 ± 2.62 | 44.78 ± 6.41 | 19.28 ± 3.69 | 21.62 ± 2.90 | 18.66 ± 3.35 |
| **TNF-α** | 15.34 ± 3.21 | 63.50 ± 7.22 | 27.01 ± 4.57 | 30.92 ± 4.92 | 26.91 ± 4.42 |
| **IFN-γ** | 1.35 ± 0.31 | 6.15 ± 1.04 | 2.97 ± 0.90 | 3.38 ± 0.49 | 2.70 ± 0.47 |
| **VEGF-α** | 25.80 ± 7.15 | 106.22 ± 21.27 | 36.83 ± 6.90 | 34.44 ± 9.06 | 26.12 ± 5.54 |
| **RANKL** | 1.99 ± 0.58 | 7.98 ± 1.28 | 3.50 ± 0.79 | 3.51 ± 0.59 | 3.02 ± 0.42 |
| **M-CSF** | 0.52 ± 0.11 | 1.87 ± 0.28 | 0.74 ± 0.21 | 0.64 ± 0.09 | 0.59 ± 0.11 |

**Supplementary Table 2:** Parameters used for sample size estimation for each assay. The table includes effect size (Δ), standard deviation (SD), type I error (α), power (1–β), statistical test used, recommended sample size per group, and actual sample size used per group.

| **Assay** | **Δ** | **SD** | **α** | **Power** | **Test** | **Recommended n/group** | **Actual n/group** |
| --- | --- | --- | --- | --- | --- | --- | --- |
| Cold sensitivity | 2 | 1.5 | 0.05 | 0.8-0.9 | Two-way ANOVA | 9-11 | 15 |
| Axial Discomfort | 20 | 15 | 0.05 | 0.8-0.9 | Two-way ANOVA | 9-11 | 15 |
| Mechanical sensitivity | 0.2 | 0.1 | 0.05 | 0.8-0.9 | Two-way ANOVA | 7-8 | 15 |
| Spinal cord *p16^ink4a^* IF | 21 | 12 | 0.05 | 0.8-0.9 | One-way ANOVA | 7-9 | 8-10 |
| Spinal cord *p16-*GFAP IF | 20 | 10 | 0.05 | 0.8-0.9 | One-way ANOVA | 5-6 | 8 |
| Spinal cord *p16-*Iba1 IF | 20 | 10 | 0.05 | 0.8-0.9 | One-way ANOVA | 5-6 | 8 |
| Spinal cord *p16-*NeuN IF | 20 | 10 | 0.05 | 0.8-0.9 | One-way ANOVA | 5-6 | 8 |
| Luminex Assay | 2-200 | 1-30 | 0.05 | 0.8-0.9 | One-way ANOVA | 4-5 | 6 |
| IVD Volume micro-CT | 0.2 | 0.05 | 0.05 | 0.8-0.9 | One-way ANOVA | 4-5 | 6-7 |
| IVD Fast staining | 1 | 0.5 | 0.05 | 0.8-0.9 | One-way ANOVA | 6-7 | 8-9 |
| IVD B-gal IF | 10 | 5 | 0.05 | 0.8-0.9 | One-way ANOVA | 5-6 | 8 |
| IVD *p16^ink4a^* IF | 10 | 5 | 0.05 | 0.8-0.9 | One-way ANOVA | 5-6 | 8 |
| IVD *p21* IF | 10 | 5 | 0.05 | 0.8-0.9 | One-way ANOVA | 5-6 | 8 |
| IVD Western blot Assay | 1 | 0.2 | 0.05 | 0.8-0.9 | One-way ANOVA | 3-4 | 6 |
| Endplate bone parameters | 15 | 5 | 0.05 | 0.8-0.9 | One-way ANOVA | 4-5 | 6 |
| Endplate B-gal IF | 10 | 5 | 0.05 | 0.8-0.9 | One-way ANOVA | 5-6 | 8 |
| Endplate *p16^ink4a^* IF | 10 | 5 | 0.05 | 0.8-0.9 | One-way ANOVA | 5-6 | 8 |
| Endplate *p21* IF | 10 | 5 | 0.05 | 0.8-0.9 | One-way ANOVA | 5-6 | 8 |
| Vertebral bone micro-CT | 5 | 2 | 0.05 | 0.8-0.9 | One-way ANOVA | 4-5 | 8 |
| Vertebral bone TRAP staining | 20 | 10 | 0.05 | 0.8-0.9 | One-way ANOVA | 5-6 | 6 |
| Vertebral bone Osteocalcin IHC | 20 | 10 | 0.05 | 0.8-0.9 | One-way ANOVA | 5-6 | 6 |
| Vertebral bone *p16^ink4a^* IHC | 20 | 10 | 0.05 | 0.8-0.9 | One-way ANOVA | 5-6 | 6 |
| Vertebral bone *p21* IHC | 20 | 10 | 0.05 | 0.8-0.9 | One-way ANOVA | 5-6 | 6 |

| **Assay** | **Spine Levels** | **number** | **sex distribution** |
| --- | --- | --- | --- |
| Cold sensitivity | n/a | 15 | 8 male, 7 female |
| Axial Discomfort | n/a | 15 | 8 male, 7 female |
| Mechanical sensitivity | n/a | 15 | 8 male, 7 female |
| Spinal cord *p16^ink4a^* IF | lumbar | 8-10 | 4-5 male, 4-5 female |
| Spinal cord *p16-*GFAP IF | lumbar | 8 | 4 male, 4 female |
| Spinal cord *p16-*Iba1 IF | lumbar | 8 | 4 male, 4 female |
| Spinal cord *p16-*NeuN IF | lumbar | 8 | 4 male, 4 female |
| Luminex Assay | L2-L5 | 6 | 3 male, 3 female |
| IVD Volume micro-CT | L2-L5 | 6-7 | 3-4 male, 3 female |
| IVD Fast staining | L2-L5 | 8-9 | 4-5 male, 4 female |
| IVD B-gal IF | L2-L5 | 8 | 4 male, 4 female |
| IVD *p16^ink4a^* IF | L2-L5 | 8 | 4 male, 4 female |
| IVD *p21* IF | L2-L5 | 8 | 4 male, 4 female |
| IVD Western blot Assay | L2-L5 | 6 | 3 male, 3 female |
| Endplate bone parameters | L2-L5 | 6 | 3 male, 3 female |
| Endplate B-gal IF | L2-L5 | 8 | 4 male, 4 female |
| Endplate *p16^ink4a^* IF | L2-L5 | 8 | 4 male, 4 female |
| Endplate *p21* IF | L2-L5 | 8 | 4 male, 4 female |
| Vertebral bone micro-CT | L2-L5 | 8 | 4 male, 4 female |
| Vertebral bone TRAP staining | L2-L5 bone | 6 | 3 male, 3 female |
| Vertebral bone Osteocalcin IHC | L2-L5 bone | 6 | 3 male, 3 female |
| Vertebral bone *p16^ink4a^* IHC | L2-L5 bone | 6 | 3 male, 3 female |
| Vertebral bone *p21* IHC | L2-L5 bone | 6 | 3 male, 3 female |

**Supplementary Table 3:** Summary of the disc or spine levels, sample size, and sex distribution per assay.

**Supplementary Table 4:** List of antibodies and dilutions used in the study.

| **Antibody** | **Source** | **Company (Catalog)** | **Dilution** |
| --- | --- | --- | --- |
| Anti-CDKN2A/p16^INK4a^ | Rabbit | Abcam (ab211542) | 1:100 |
| Anti-p21 | Rabbit | Abcam (ab188224) | 1:500 |
| Anti-GFAP | goat | Millipore Sigma (SAB2500462) | 1:500 |
| IBA1 Monoclonal | Mouse | Invitrogen (MA5-27726) | 1:100 |
| Anti-NeuN purified | guinea pig | Millipore Sigma (ABN90P) | 1:500 |
| NRF-2 | Rabbit | ABclonal (A0674) | 1:1000 |
| Phospho-NF-kB p65 | Rabbit | ABclonal (AP0475) | 1:2000 |
| NF-kB p65 | Rabbit | ABclonal (A19653) | 1:5000 |
| p53 | Rabbit | ABclonal (A25915) | 1:8000 |
| MDM2 | Rabbit | ABclonal (A0345) | 1:500 |
| p21 | Rabbit | ABclonal (A19094) | 1:1000 |
| Osteocalcin/OCN | Rabbit | ABclonal (A6205) | 1:100 |
| HRP-conjugated Goat anti-Rabbit IgG  (H+L) | Goat | ABclonal (AS014) | 1:5000 |
| Goat Anti-Rabbit IgG H&L (HRP) | Goat | Abcam (ab6721) | 1: 5000 |
| Goat anti-Rabbit IgG (H+L) Cross-Adsorbed, Cyanine3 | Goat | Invitrogen (A10520) | 5 µg/mL |
| Donkey anti-Rabbit IgG (H+L) Highly Cross-Adsorbed Secondary Antibody, Alexa Fluor™ 647 | Donkey | Invitrogen (A31573) | 5 µg/mL |
| Donkey anti-mouse IgG (H+L) Cross-Adsorbed Secondary Antibody, Alexa Fluor™ 594 | Donkey | Invitrogen (A21203) | 5 µg/mL |
| Alexa Fluor® 488 Donkey Anti-Rabbit IgG (H+L) | Donkey | Jackson Immuno (711-545-152) | 1:2000 |
| Alexa Fluor® 647 Goat Anti-Guinea Pig IgG (H+L) | Goat | Jackson Immuno (106-605-003) | 1:2000 |
| Cy™3 Donkey Anti-Goat IgG (H+L) | Donkey | Jackson Immuno (705-165-003) | 1:2000 |
